# Phage receptor binding protein and Fc fragment fusion enhances phagocytosis of *Y. enterocolitica*

**DOI:** 10.1186/s13568-025-01948-9

**Published:** 2025-09-26

**Authors:** Karolina Filik-Matyjaszczyk, Irwin Matyjaszczyk, Marzena Ciesielska, Bożena Szermer-Olearnik, Krzysztof Mikołajczyk, Andrzej Gamian

**Affiliations:** 1 Hirszfeld Institute of Immunology and Experimental Therapy, Polish Academy of Sciences, 53-114 Wroclaw, Poland; 2 Department of Biochemistry, Selvita S.A, 30-394 Cracow, Poland

**Keywords:** *Yersinia Enterocolitica*, RBP, Phagocytosis, Immunotherapy, Macrophages, Neutrophils

## Abstract

Bacteriophage receptor binding proteins (RBPs) belong to a group of proteins that are components of the bacteriophage tail. RBPs are mainly responsible for recognizing receptors on the bacterial host surface and enabling infection and subsequent progeny phage multiplication. Our previous research suggested that recombinant tail fiber gp17 (TFPgp17) protein from *Yersinia enterocolitica* phage φYeO3-12 recognizes serotype O:3 with high specificity and could be used as a diagnostic tool. In this paper, we study the possibility of using modified TFPgp17 in fusion with IgG1 Fc immunoglobulin fragment (Fc_TFPgp17) to improve the efficacy of pathogen recognition by phagocytic cells, opsonization of bacterial cells, and then phagocytosis by the THP-1 macrophages and the HL-60 neutrophils. We demonstrate that the Fc_TFPgp17 protein can bind both bacterial and phagocytic cells, due to the presence of the RBP domain and the IgG1 Fc fragment, respectively. Additionally, it contributes to the better recognition of bacterial cells and their subsequent phagocytosis by phagocytes. Additionally, we proved that glycosylation of Fc_TFPgp17 which occurred during protein production, harms the ability to recognize *Y. enterocolitica* O:3. Fc_TFPgp17 is a bispecific protein that possesses two biological functions: (I) recognized *Y. enterocolitica* O:3 cells via phage RBP (TFPgp17) and (II) recognized phagocytic cells via Fc-fragment IgG1 immunoglobulin. In this work, we demonstrated that the recombined bispecific protein facilitates more effective recognition and opsonization of the pathogenic bacterial cell, leading to its subsequent phagocytosis.

**Supplementary Information:**

The online version contains supplementary material available at 10.1186/s13568-025-01948-9.

## Introduction

*Yersinia enterocolitica* is an etiological cause of yersiniosis – a foodborne, zoonotic infectious disease caused by pathogenic *Y. enterocolitica* and *Yersinia pseudotuberculosis* (Shoaib et al. 2019). Yersiniosis is a self-limiting gastroenteritis, with symptoms that mostly include self-limiting diarrhea, weight loss, severe abdominal pain, dehydration, bloody feces, and, in serious cases, septicemia. The primary sources of this bacterium are animal-originated foods, mostly pork, and the infection is caused by eating raw or undercooked pork and dairy products contaminated by the bacteria. Consequently, pigs are the main reservoir of this pathogen (Drummond et al. 2012; Rakin et al. 2015; Triantafillidis et al. 2020). Data presented in the Annual Epidemiological Report from 2022 indicated that yersiniosis was the fourth most commonly reported gastrointestinal infection in the EU/EEA after campylobacteriosis, salmonellosis, and Shiga toxin-producing *Escherichia coli* (STEC). A total of 8,037 confirmed cases of yersiniosis (caused by *Y. enterocolitica* and *Y. pseudotuberculosis*) were reported by 27 EU/EEA countries, with an overall rate of 2.2 cases per 100,000 population (European Centre for Disease Prevention and Control (ECDC) 2022). According to previous research by Wielkoszyński et al., 2018, the infection rate could be much higher than present, because only serious infections are registered, so mild infections may be overlooked (Wielkoszynski et al. 2018).

Receptor binding proteins (RBPs) are a group of bacteriophage proteins that determine phage infectivity by binding to receptors on the host cell surface, in the first step of the infection process. The group consists of two types of proteins depending on their morphology: Tail fiber proteins (TFP) and Tailspikes (TS) (De Jonge et al. 2019; Witte et al. 2021). RBPs may exhibit enzymatic activity toward saccharide structures and specifically bind receptors on bacterial cell surfaces (Dunne et al. 2021). RBPs recognize a variety of receptors which can be divided into two groups: protein-derived receptors, such as porins or flagella, and saccharide-derived receptors, which include components of bacterial outer membranes, LPS, or capsules (Witte et al. 2021). Recent years have seen a growing interest in the study of RBPs and their practical use in bacterial pathogen diagnostics. Numerous studies have demonstrated that RBPs hold significant promise as diagnostic tools, potentially improving diagnostics and replacing traditional microbial identification methods that rely on culture and biochemical testing (Denyes et al. 2017; Filik et al. 2022a; Filik et al. 2022b; Javed et al. 2013; Klumpp et al. 2023; Lee et al. 2020; Santos et al. 2020; Sumrall et al. 2020).

Since the 1940s, when penicillin was first discovered, antibiotics have dominated antibacterial research for the next sixty years, as small-molecule antibiotics were the best-studied approach for controlling bacterial infections in humans and animals with low cost and good efficacy (Lewis 2013). Numerous strategies exist to treat infections caused by antibiotic-resistant bacterial pathogens. While traditional development strategies continue to dominate the research for solutions to antimicrobial resistance, some scientists are exploring biological approaches as potential breakthroughs. One such method involves the use of monoclonal antibodies (mAbs) in therapy. Although antibodies have been extensively studied and successfully used in the anti-cancer, autoimmune, and antiviral fields, the development of mAbs for bacterial infections is still in its infancy. For bacterial infections, there is untapped potential and a substantial lack of knowledge regarding mAbs and their possible prophylactic or therapeutic use. Designing and producing antibodies that specifically recognize bacterial cells and enhance the immune system response is challenging. mAbs may exhibit antimicrobial activity against pathogens through many mechanisms, such as bactericidal action, direct action on the biofilm, blockage of iron uptake and adhesion, as well as mediating opsonophagocytosis and the antibacterial activity of the complement system and serum (Zurawski and McLendon 2020).

According to a review by Zurawski and McLendon 2020; from 2002 to 2020, 40 mAbs were approved by the Food and Drug Administration (FDA) for the treatment of various diseases, but none were developed for bacterial infection. Surprisingly, there are no mAbs under development for bacterial infections; however, the authors presented 14 mAbs that were subjected to clinical trials in the past. Past failures may have diminished interest in pursuing mAb strategies against bacterial pathogens. The primary focus of past research has been on *Staphylococcus aureus* and *Pseudomonas aeruginosa*. One example, tefimazumab, a mAb for the treatment of *S. aureus* infections, was effective in animals but failed in phase 2 of the clinical trial. Similarly, KB001-A, developed by KaloBios against *P. aeruginiosa*, was effective in the animal model and proven safe for use in humans, however, it was not effective in phase 2 clinical trials on patients under mechanical ventilation (Baer et al. 2009; Jain et al. 2018; Sause et al. 2016; Zurawski and McLendon 2020). Several factors contributed to these failures: many studies focused on mAb specificity, designing a single antibody for one target on the bacterial cell, when there are around 100 targets available on the bacterial surface. Moreover, serious bacterial infections are often caused by multiple species. Additionally, bacterial lifestyle – surface protein profile, biofilm production, and capsule production, should be considered in mAb design. Despite the challenges in designing mAbs, their use offers several benefits, such as (I) minimal toxicity, (II) longevity (IgG half-life is around 21 days (Hey 2016), (III) specificity (not disrupt the commensal bacteria), (IV) rapid killing via multiple mechanisms (direct killing, neutralization, complement deposition, and opsonization), which also limits toxic shock by suppressing inflammation, and (V) the potential to reduce the spread of antibiotic resistance and exhibit additive or synergistic effect when co-administered with antibiotics (Dunn-Siegrist et al. 2007; Heesterbeek et al. 2018; Hey 2016; Wagner and Maynard 2018; Zurawski and McLendon 2020).

One of the first experimental approaches to utilize bacteriophage proteins in antibody development was the creation of “lysibodies” by Raz et al. The authors designed a two-chain homodimer consisting of an Fc fragment of IgG1 immunoglobulin and a binding domain of phage lysins. These modular enzymes combine high-affinity binding to carbohydrate determinants on the bacterial surface and cleavage activity (Raz et al. 2017). Scientists performed several experiments demonstrating that lysibody can improve phagocytosis by macrophages and neutrophils, functioning as an opsonin and leading to pathogen elimination. Moreover, they showed that lysibody promoted complement deposition on the pathogen surface, leading to efficient removal by phagocytes. Importantly, in vivo experiments on mice confirmed the efficacy and protective nature of the presented approach. This strategy opens new avenues for addressing long-standing challenges and creating high-affinity mAb specifically binding the bacterial cell wall carbohydrates (Raz et al. 2017). The authors highlight the advantages of this approach, emphasizing the ability to bind to carbohydrate targets on the bacterial wall surface, which are highly conserved and often crucial for maintaining cell wall functionality. The lysins have been optimized by evolution and are a conserved group of proteins that do not mutate often, as non-functional lysins produced by bacteriophages would hinder their proliferation (Raz et al. 2017). The production of anti-lysibody antibodies may lead to the development of side effects during the therapy, however, the authors suggest that in this case, the use of lysibodies could be restricted to treat life-threatening infections, either alone or in combination with conventional therapies such as antibiotics. Due to their properties, lysibodies can enhance the immune response by allowing phagocytic cells to recognize the pathogen more quickly and efficiently (Raz et al. 2017, 2018).

In our previous study by Filik et al. 2022a, we focused on bacteriophage tail proteins and demonstrated that the TFPgp17 protein from the *Y. enterocolitica* bacteriophage φYeO3-12 recognizes the O:3 serotype of this pathogen with high specificity compared to other bacterial strains used in this research. We validated our hypothesis using ELISA and Transmission Electron Microscopy (TEM) (Filik et al. 2022a). Based on these results, we designed a fusion protein with dual nature: TFPgp17 fused with the Fc fragment of IgG1 (Fc_TFPgp17) to test the efficiency of the bacterial phagocytosis in its presence. Since TFPs are naturally responsible for recognizing bacteria, playing the most important role in phage infection, their use to create Fc fusion proteins simulating monoclonal antibodies, which act as opsonins, is highly rational and promising. The main objective of the research we presented was to assess the efficacy of Fc_TFPgp17 in bacterial recognition, phagocytosis, and immune modulation.

## Methods

### Bacterial strain and growth conditions

*Yersinia enterocolitica* 6471/76-c (O:3 serotype) strain (HER 1249; Felix d’Herelle Reference Center for Bacterial Viruses) was used in this work and was obtained from Professor Mikael Skurnik (Pajunen et al. 2000; Skurnik 1984). Bacteria were grown in LB medium (Luria-Bertani) at 28 °C at 120 rpm.

### Cell cultures and growth conditions

Human leukemia monocytic cell line THP-1 and human acute promyelocytic leukemia cell line HL-60 were grown in RPMI 1640 medium (Roswell Park Memorial Institute) supplemented with 10% heat-inactivated FBS (Fetal Bovine Serum) (Invitrogen) at 37 °C in the presence of 5% CO_2_.

### THP-1 differentiation

THP-1 cell line (ATCC; TIB 202) was differentiated to macrophages with a distinct M (IL-4) phenotype according to protocol adapted from Baxter et al. 2020. 5 × 10^6^ cells were seeded into a 25 cm^2^ culture bottle in 5 ml antibiotic-free RPMI 1640 media supplemented with 10% heat-inactivated FBS (Invitrogen) and 5 µg/ml PMA (Phorbol 12-myristate 13-acetate) (Sigma). Cells were incubated for 24 h at 37 °C, in 5% CO_2_. Next, PMA was removed, and cells rested for 72 h. In the next step, cells were stimulated with 20 ng/ml IL-4 (Bio-techne) to obtain the IL-4-like macrophage phenotype (Baxter et al. 2020). After every step, cells were analyzed in light microscopy for changes in morphology. After differentiation, cells were stained with the Giemsa dye to check the phenotype (data not shown). FCγR (anti-CD16-APC, R&D RDFAB25461; anti-CD32-FITC, BD 555448; anti-CD64-PeCy7, BD 561191) expression in the cells was verified via flow cytometry (Supplementary materials).

### HL-60 differentiation

The HL-60 cell line (ATCC; CCL 240) was differentiated into neutrophils according to Gee et al. 2012 with slight modifications. 5 × 10^6^ HL-60 cells were seeded onto a 25 cm^2^ culture bottle in 5 ml antibiotic-free RPMI 1640 media supplemented with 10% heat-inactivated FBS (Invitrogen). Cells were resuspended in a full RPMI 1640 medium with 1.25% DMSO to induce differentiation. Cells were incubated for 96 h at 37 °C, 5% CO_2_ (Gee et al. 2012). After each step, cells were analyzed in light microscopy for changes in morphology. Following differentiation cells were stained with the Giemsa dye to check the phenotype (data not shown). FCγR (anti-CD16-APC, R&D RDFAB25461; anti-CD32-FITC, BD 555448; anti-CD64-PeCy7, BD 561191) expression in cells was verified via flow cytometry (Supplementary materials).

### Recombinant protein production in CHO cells

Fc-fused RBP was obtained from Biointron Company. Proteins were produced in the CHO mammalian expression system. Then, proteins were purified using 3 steps of purification: Protein A affinity chromatography, Ni-NTA affinity chromatography, and size exclusion chromatography. According to Biointron’s “General protocol for expression and purification,” during all the steps of purification, proteins were analyzed using SDS-PAGE, and concentration was measured by NanoDrop (Supplementary materials). In the last step, the endotoxin levels were evaluated.

### Removal of N-glycans using PNGase F

Digestion of Fc_TFPgp17 by PNGase F was performed in native conditions. Briefly, 10 µl of 0.5 M ammonium bicarbonate buffer pH 7.8 was added to the 1 ml of Fc_TFPgp17 solution (0.72 mg of protein). The deglycosylation reaction was carried out with 500 units of PNGase F (Promega, Madison, WI) for 18 h at 37 °C. After incubation, the product was analysed by SDS-PAGE (Mikolajczyk et al. 2021).

### Fc_TFPgp17 binding to the bacterial cells studied by ELISA

For the bacterial cell-based sandwich ELISA, Filik et al. (2022a) protocol was used with minor modifications. *Y. enterocolitica* 6471/76-c bacteria strain was used in the experiments. In the first step, 5 ml of bacterial culture in LB medium was incubated overnight (O/N) at 28 °C. The next day, bacterial cultures were refreshed and incubated at 28 °C with shaking at 120 rpm until the OD600 reached 0.4. When the OD600 reached the appropriate value, the cultures were washed with PBS-T (Phosphate Buffered Saline; PBS supplemented with 0.1% Tween 20) by centrifugation (2000 g, 5 min) to remove the medium. The bacterial pellet was resuspended in 5 ml of fresh PBS and diluted tenfold. 200 µl per well of the bacterial suspension was added to Maxisorp 96-well microplates, keeping O/N at 4 °C, and then the plate was centrifuged (600 g, 4 °C, 20 min). The plate was fixed by adding 200 µl of 0.1% glutaraldehyde per well and incubating for 30 min at room temperature (RT). After incubation, the solution was discarded. Next, 200 µl of 0.1% BSA (Bovine Serum Albumin)/PBS solution supplemented with 0.1 M glycine was added to wells, and the plates were incubated at RT for 2 h. After removing the solution was discarded, 200 µl of 4% (w/v) BSA (BSA Blocker, Thermo Scientific) blocking solution was added to each well, and plates were incubated at RT for 2 h. Plates were washed 3X with PBS-T and then 50 µl solution of tested proteins (Fc_TFPGp17, Fc_control, and Fc_TFPGp17, Fc_control de-N-glycosylation) was added to wells in concentrations ranging from 0.0156 µM to 1.5 µM, plates were incubated at RT for 2 h. Plates were washed 3X and 50 µl of HRP-Rabbit Anti-Human IgG, Fcγ Antibody (Peroxidase AffiniPure Rabbit Anti-Human IgG, Fcγ fragment specific; Jackson), diluted 1:10.000 in blocking buffer, was added to the wells, and the plates were incubated at RT for 60 min. Plates were washed 3X with PBS-T and incubated with TMB substrate (100 µl per well) (Thermo Scientific) at RT for 5–15 min. The reaction was stopped by adding 100 µl of 0.18 M sulfuric acid. Absorbance was measured at 450 nm on a microplate reader (Tecan Spark) (Filik et al. 2022a).

### Fc_TFPgp17 binding to the THP-1-derived macrophages and HL-60-derived neutrophils was studied using flow cytometry

THP-1 and HL-60 cells were differentiated as described above. The Fc-fusion proteins were labeled with Zenon™ Human IgG Labelling Kit (Invitrogen). The labelling was performed according to the manufacturer’s instructions: for 1 µg of antibody/protein, 5 µl of the Zenon™ human IgG labelling reagent (Component A) was used, and the mixture was incubated for 5 min. Next, 5 µL of the Zenon™ blocking reagent (Component B) was added to the mixture and incubated for the next 5 min. After labeling, serial dilutions of conjugated Fc-fused proteins were prepared in PBS, with concentrations ranging from 0.25 µM to 0.000475 µM. THP-1 and HL-60 cells were counted under a microscope and placed into FACS tubes (5 × 10^5^ cells per tube). Dilutions of conjugated Fc-fusion protein were added to the cells, and the mixture was incubated for 30 min at RT in the dark. Following incubation, the cells were washed once with sterile PBS with 2% of BSA, resuspended in PBS, next samples were acquired on the flow cytometer. The analysis was performed using the LSR Fortessa flow cytometer in the FITC channel (498/517 nm) with Diva software (BD Biosciences).

### Phagocytosis assay using flow cytometry

Firstly, a 5 ml culture of *Y. enterocolitica* 6471/76-c in LB medium was incubated at 28 °C O/N. Then, bacterial cultures were refreshed and incubated at 28 °C with shaking at 120 rpm until the OD600 reached 0.1. One ml of bacteria culture was transferred to an Eppendorf tube, and cells were washed once with 1 ml of sterile PBS (3000 g, 5 min) and resuspended in 500 µl of sterile PBS. For phagocytosis measurement by flow cytometry, bacterial cells were stained with pH Rodo Green Ester (Thermo Scientific): 2.5 µl of the 10 mM dye was added to 500 µl of the bacterial suspension. The suspension was incubated for 60 min with slow shaking. Next, the reaction was stopped by adding 1 M Tris-HCl pH 7.5 to a final concentration of 10 mM (5 µl from stock), cells were washed once with PBS to remove excess dye.

THP-1 and HL-60 cells were differentiated according to the protocol described above. After differentiation, the cells were collected by centrifugation (1100 rpm/175 g, 5 min), washed once in RPMI 1640 medium without FBS, and resuspended in the same medium. Next, cells were counted under a microscope in the Burker chamber and diluted to an appropriate concentration (2 × 10^5^ cells per well). In the next step, bacterial cells were opsonized with Fc_TFPGp17 and Fc_control (total volume in the well at this stage was 50 µl) on a non-treated U-shaped 96-well plate. The bacterial cells were incubated with proteins in a concentration range of 0.0156 µM to 1.5 µM diluted in RPMI 1640 without FBS for 30 min at 37 °C at 300 rpm on a plate shaker (Biosan). After incubation, 50 µl of differentiated THP-1 or HL-60 cells (2 × 10^5^ cells/well) were added to the opsonized bacteria at a ratio of 10:1. The mixed cultures were incubated for 60 min at 37 °C under 5% CO_2_. After incubation, the cells were washed two times with sterile PBS, transferred from the plate to FACS tubes, and fixed with 1% paraformaldehyde for 15 min at 4 °C. Cells were washed once with sterile PBS, resuspended in 200 µl of PBS, and read using an LSR Fortessa flow cytometer with Diva software 8.2 (BD Biosciences). Flow cytometer performance, stability, and data reproducibility were sustained and checked in real-time by using the CST BD Lot 23,344 quality control. Data was then analyzed using FlowJo version 10.

### Quantification of viable intracellular bacteria (Gentamicin Protection Assay)

**Fig. 1:**
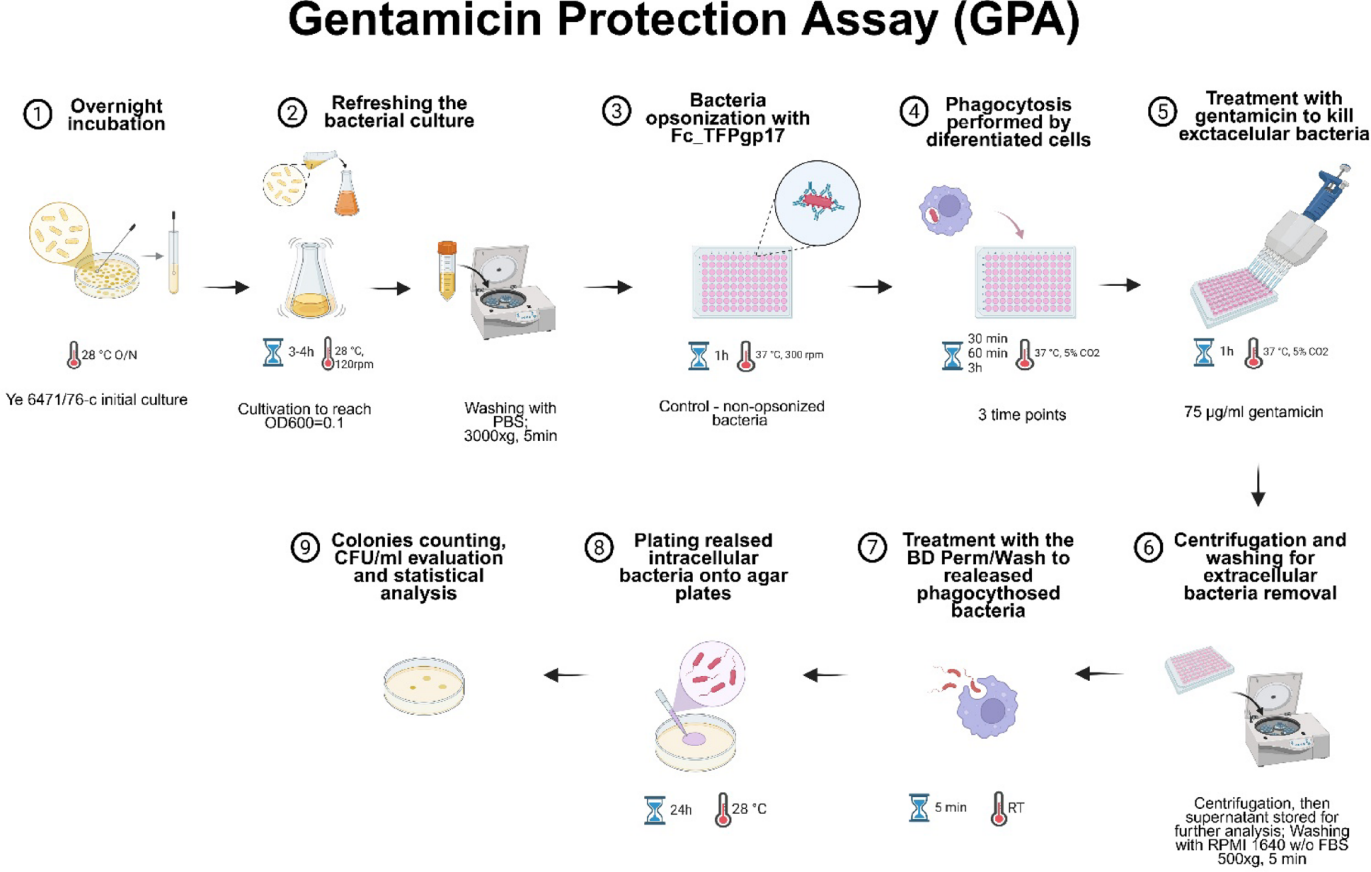
Gentamicin Protection Assay pipeline. The experiment was performed in three time points: 30 min, 60 min and 3 h to complement the results obtained from flow cytometry analysis of opsonophagocythosis. Created with BioRender.com.

Firstly, a 5 ml culture of *Y. enterocolitica* 6471/76-c in LB medium was incubated O/N at 28 °C, refreshed, and incubated at 28 °C with shaking at 120 rpm until the OD600 reached 0.1. THP-1 and HL-60 cells were differentiated according to the protocol described earlier. Differentiation cells were collected by centrifugation (1100 rpm/175 g, 5 min), washed once in RPMI 1640 medium without FBS, and resuspended in the same medium. Next, cells were counted under a microscope in the Burker chamber and diluted to an appropriate concentration (2 × 10^5^ cells per well). Cells were incubated for 2 h to induce starvation, and during this process, 1 ml of bacterial culture was washed once with 1 ml of sterile PBS (3000 g, 5 min). Next, the bacterial cells were resuspended in 500 µl of sterile PBS and opsonized with Fc_TFPgp17 on a non-treated U-shaped 96-well plate at 1 µM final protein concentration (diluted in RPMI 1640 without FBS). Samples were incubated for 30 min at 37 °C at 300 rpm on a plate shaker (Biosan). After opsonization, 2 × 10^5^ of differentiated and starved THP-1 or HL-60 were added to the opsonised bacteria at a ratio of 10:1. The mixed cultures were incubated from 30 min to 3 h at 37 °C under 5% CO_2_. At each time point (30 min, 60 min, and 3 h), RPMI 1640 without FBS, supplemented with gentamicin, was added to each well to achieve a final concentration of 75 µg/ml, and samples were incubated for 1 h at 37 °C, 5% CO_2_. After the plates were centrifuged (500 g, 5 min), the supernatants were collected and stored at -20 °C for further analysis. The cells were washed once with RPMI 1640 w/o FBS to remove gentamicin and extracellular (unbound) bacteria, resuspended in 300 µl of Perm/Wash solution (BD), and incubated for 5 min at RT. Then, serial dilutions were prepared from each well and placed onto agar plates for CFU (Colony Forming Units) determination. The modified protocol comes from a publication by Sharma and Puhat, 2019 and Biryukov et al. 2023 (Biryukov et al. 2023; Sharma and Puhar 2019). The experiment main steps are showed on the Fig. 1.

### Measurement of the quantities of selected cytokines

Cytokine levels were measured using commercially available ELISA kits: IL-10 (BMS215-2, Invitrogen) and TNF-α (KHC3011, Invitrogen). All ELISA tests were performed according to the manufacturer’s instructions. The supernatants used for these tests were obtained from the GPA. Supernatants were stored at −20 °C.

### Statistical analysis

Data analysis and preparation of graphs were performed using GraphPad Prism version 7 (GraphPad Software Inc., San Diego, CA, USA. Statistical significance analysis was performed using an unpaired Student t-test with Welch corrections.

## Results

### The protein produced in CHO cells with N-glycosylation and Fc fragment affects the efficiency of its binding to Y. enterocolitica 6471/76-c

The recombinant bacteriophage TFPgp17 protein fused with the IgG1 Fc fragment (referred to as Fc_TFPgp17) and the Fc fragment used as a control (referred to as Fc_control) were obtained from Biointron company. The proteins were designed for their production in a mammalian expression system to ensure compatibility with subsequent experiments involving human cell lines THP-1 and HL-60. The Fc fragment of the human IgG1 immunoglobulin is glycosylated in the post-translational modification process, which is essential for its efficient binding to Fcγ receptors present in immune cells. In contrast, phage proteins are typically non-glycosylated due to the nature of their synthesis in bacterial hosts, although they may contain sequence motifs that are potential sites for glycosylation Fig. 3.

**Fig. 2:**
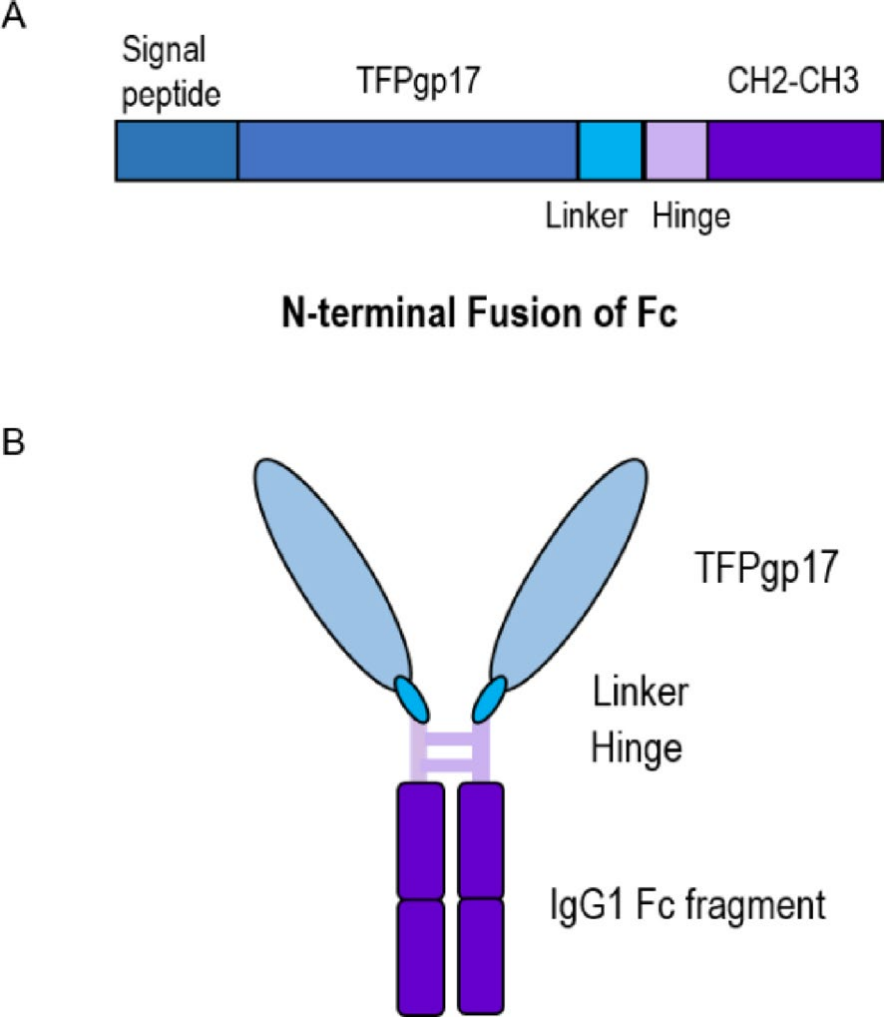
Graphical presentation of the Fc-fused phage TFPgp17 protein. The designed protein is composed of two components and mimics antibodies: the Fc fragment of a human IgG1 immunoglobulin and the TFPgp17 phage adhesin protein (1B). The TFPgp17 protein, responsible for specific recognition of bacterial surface receptors, is fused via the N-terminal region with the IgG1 Fc fragment containing CH2 and CH3 domains (1 A), which bind to Fcγ receptors on the surface of immune cells, facilitating effector functions

Figure 2 shows the structure of the recombinant protein, highlighting the fusion of the Fc fragment of the IgG1 immunoglobulin to the RBP of the bacteriophage at the N-terminus. According to the literature, the RBPs of bacteriophages exhibit an N-terminal domain structure responsible for the attachment of the protein to the tail structure, while the C-terminal is responsible for the specific recognition of receptors on the bacterial host cell surface (Pajunen et al. 2001; Pyra et al. 2020).

**Fig. 3:**
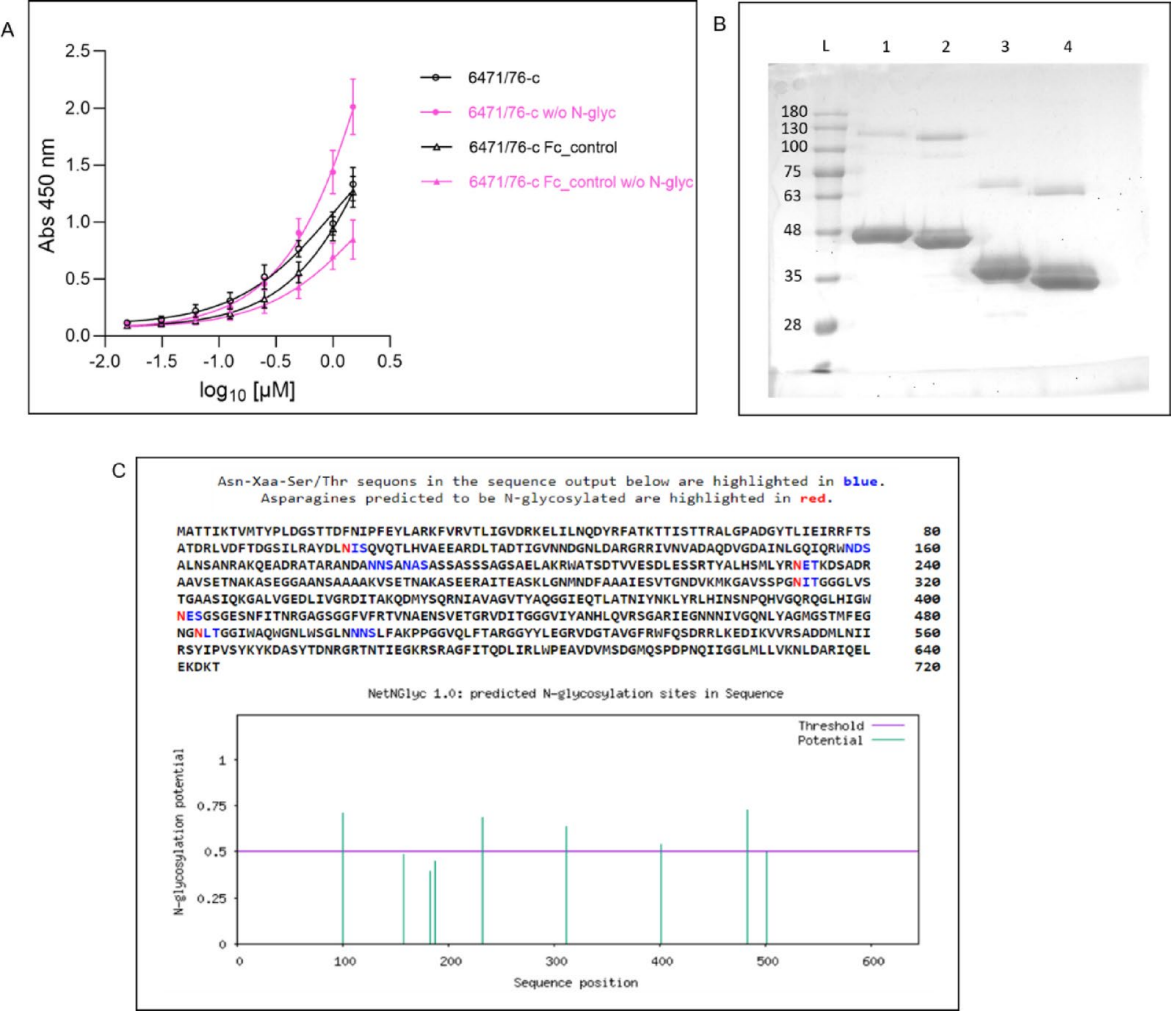
**A** N-glycosylation influences the ability of TFPgp17 to bind *Y. enterocolitica* 6471/76c. The graph presents the results of an ELISA test evaluating the ability of Fc_TFPgp17 (tested concentration of protein ranging from 0.0156 µM to 1.5 µM), produced in the CHO expression system, to recognize *Y. enterocolitica* 6471/76c, serotype O:3. The results are displayed in pink for the protein with N-glycans removed by PNGase F and in black for the protein with intact N-glycans. An IgG1 Fc fragment was used as a control. The graph represents data from two biological replicates, with each experiment performed in two technical replicates. **B** SDS-PAGE analysis of de-N-glycosylated Fc_TFPgp17 protein. Reducing conditions. L – molecular weight ladder (BlueStar Prestained Protein Marker, Nippon Genetics), 1 – Fc_TFFgp17, 2 – Fc_TFPgp17 treated with PNGase F, 3 – Fc_Control, 4 – Fc_control treated with PNGase F, **C** Analysis of potential N-glycosylation sites. NetNGlyc-1.0 software was used to predict N-glycosylation sites in the protein primary sequence. For the analysis, the TFPgp17 sequence from UniProt was taken (UniProt ID Q9T0Z9). Five potential sites of N-glycosylation were predicted (Asparagine residues presented in red) (Gupta 2002)

The first step of the experimental process was the ELISA test with the whole bacterial cell to confirm the specificity and ability of the Fc_TFPgp17 protein to bind the *Y. enterocolitica* 6471/76-c serotype O:3 (Fig. 3A). According to our previous results, TFPgp17 is characterized by high specificity towards *Y. enterocolitica* O:3 strains, and fusion with MBP-his did not affect the binding of the bacterial cells. In the paper by Filik et al. 2022, we checked the specificity on 13 *Y. enterocolitica* strains (different serotypes) and additionally on 4 Gram-negative strains (2 *E. coli*, *P. aeruginosa*, and *E. aerogenes*). Moreover, our negative control was a spontaneous R1 mutant of *Y. enterocolitica*, which is characterized by a lack of O-antigen and helps us to prove that O-antigen is a key receptor for TFPgp17. It is known from the literature that the receptors on the surface of bacteria, to which TFPgp17 attaches, are often sugar residues located in the O-specific chain of LPS (Filik et al. 2022a). In this paper, ELISA was performed to confirm that the Fc domain does not affect the binding of Fc_TFPgp17 to bacterial cells. The obtained results indicate that Fc fusion does not hinder the binding of Fc_TFPgp17 to the tested *Y. enetrocolitica* strain (Fig. 3A).

Additionally, as is well known, recombinant proteins produced in eukaryotic expression systems often undergo a glycosylation process, which is absent in bacteria. However, phage proteins may still contain motifs in their sequences that could undergo glycosylation under appropriate conditions. According to analysis in the NetNGlyc-1.0 software, TFPgp17 contains several N-glycosylation sites in its sequence (Fig. 3C).

In our study, we confirmed that N-glycosylation can occur in the TFPgp17 protein and may affect its binding to bacterial cells. The ELISA results obtained for Fc_TFPgp17 containing N-glycans, compared to Fc_TFPgp17 where N-glycans were enzymatically removed with PNGase F (Fig. 3B), show higher absorbance for the PNGase F treated samples. This finding indicates better binding of the non-glycosylated protein and suggests that glycosylation may hinder binding to bacterial cells.

Moreover, ELISA results show that Fc_control also binds to the tested bacterial strains, although with lower strength compared to Fc_TFPgp17. This observation indicates either non-specific binding or the presence of immunoglobulin binding proteins on the surface of the tested *Y. enterocolitica* strains.

### Fc_TFPgp17 can recognize and bind phagocytic cells

The next step in the research was to confirm the binding of the proposed bispecific protein Fc_TFPgp17 to THP-1 and HL-60 human cell lines, differentiated into macrophages and neutrophils, respectively. For this purpose, flow cytometry analysis, using the Zenon IgG Labelling reagent and measuring MFI (Mean Fluorescence Intensity), was performed.

**Fig. 4:**
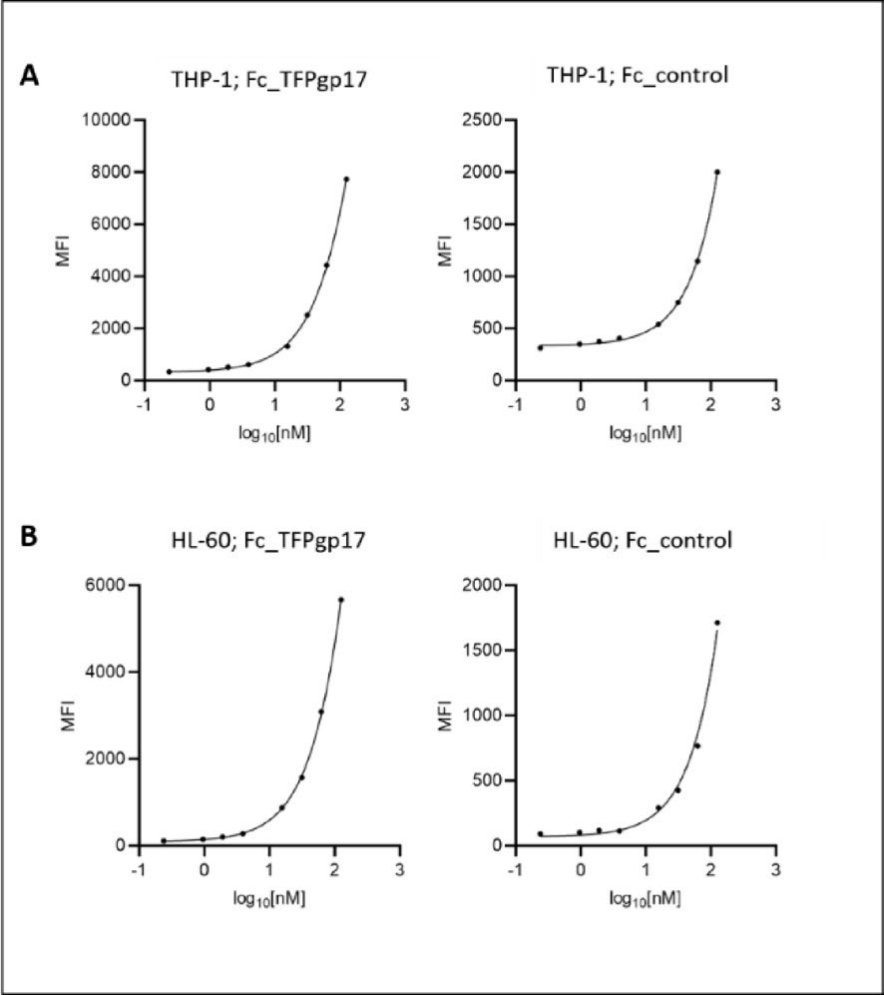
Fc_TFPgp17 recognizes the THP-1-derived macrophages and the HL-60-derived neutrophils via the Fc fragment of the Fc-fused protein. **A** binding to the THP-1-derived macrophages, **B** binding to the HL-60-derived neutrophils studied using flow cytometry and Zenon Labeling Kit for antibody labeling. In the experiment, Fc_TFPgp17 and Fc_control were used in concentrations ranging from 0.000475 µM to 0.25 µM

Binding was confirmed for Fc_TFPgp17 and Fc_control in both cell lines (Fig. 4).

However, the correct binding curve could not be obtained due to the high concentration of bispecific protein required to achieve saturation or non-specific binding.

### Fc_TFPgp17 acts as an opsonin and enhances the phagocytosis of bacterial cells compared to untreated controls in an in vitro model tested by flow cytometry

**Fig. 5:**
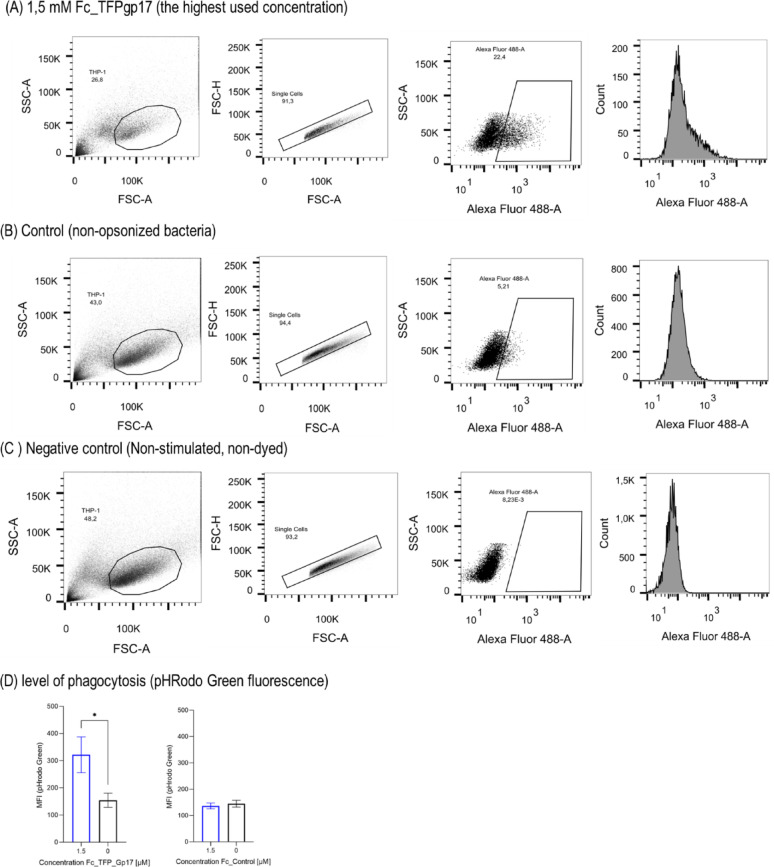
Fc_TFPgp17 acts as an opsonin in the THP-1 cell line phagocytosis assay using pHRodo Green Dye and flow cytometry. The phagocytosis of *Y. enterocolitica* 6471/76-c cells opsonized with 1.5 µM concentrations of Fc_TFPgp17 compared to non-opsonized cells. The Fc fragment, without TFPgp17, was used as a control. Gating strategy for flow cytometry analysis: macrophages were first gated based on forward scatter (FSC) and side scatter (SSC) properties (FSC-A/SSC-A), then single cells FSC-A/FSC-H, followed by the identification of highly fluorescent macrophages at a statistically significant Fc_TFPgp17 concentration. **A** The highest used concentration of Fc_TFPgp17 for opsonization of bacteria – 1.5 µM, **B** Control – phagocytosis performed on no-opsonized bacteria, **C** Control – non-stimulated and not dyed THP-1 cells, **D** The column graphs show the level of phagocytosis in the highest used concentration of Fc_TFPgp17 used for opsonization compared to the Fc-fragment used for opsonization. The graph represents data from 2 biological replicates. Statistical significance analysis using an unpaired t-test with Welch corrections was performed for the relevant samples. (*-*P* ≤ 0.05)

**Fig. 6:**
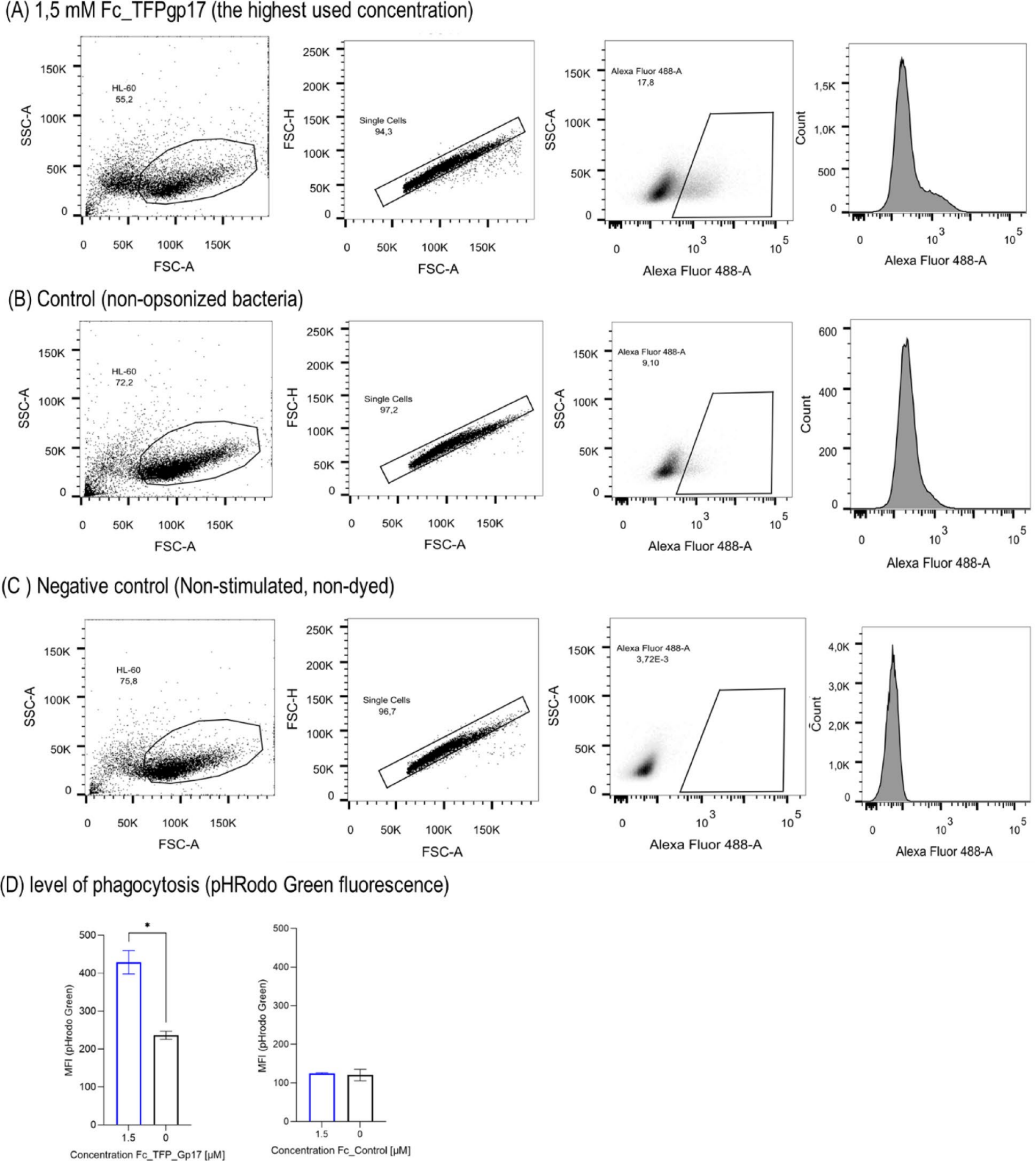
Fc_TFPgp17 acts as an opsonin in the HL-60 cell line phagocytosis assay using pHRodo Green Dye and flow cytometry. The phagocytosis of *Y. enterocolitica* 6471/76-c cells opsonized with 1.5 µM concentrations of Fc_TFPgp17 compared to non-opsonized cells. The Fc fragment, without TFPgp17, was used as a control. Gating strategy for flow cytometry analysis: macrophages were first gated based on forward scatter (FSC) and side scatter (SSC) properties (FSC-A/SSC-A), then single cells FSC-A/FSC-H, followed by the identification of highly fluorescent macrophages at a statistically significant Fc_TFPgp17 concentration. **A** The highest used concentration of Fc_TFPgp17 for opsonization of bacteria – 1.5 µM. **B** Control – phagocytosis performed on no-opsonized bacteria. **C** Control – non-stimulated and not dyed THP-1 cells. **E** The column graphs show the level of phagocytosis in the highest used concentration of Fc_TFPgp17 used for opsonization compared to the Fc-fragment used for opsonization. The graph represents data from 2 biological replicates. Statistical significance analysis using an unpaired t-test with Welch corrections was performed for the relevant samples. (*-*P* ≤ 0.05)

Fc_TFPgp17 is a bispecific protein that binds to phagocytic cells via the Fc-fragment and to bacterial cells via the RBP molecule. In the next step, we performed flow cytometry to assess the efficiency of phagocytosis in the presence of Fc_TFPgp17 in THP-1-derived macrophages and HL-60-derived neutrophils (Figs. 5 and 6). To test phagocytosis, flow cytometry and labelling of the bacteria with pHRodo Green dye were performed. pHRodo Green dye fluoresces at low pH, indicating that the labelled bacteria are inside the phagosomes of phagocytic cells. The use of this dye enabled the elimination of non-specific signals from within the cells and allowed for the measurement of specific signals from the intracellular bacteria present in the phagosomes.

We used Fc_TFPgp17 as an opsonin and tested whether there were statistically significant differences in phagocytosis between bacterial cells opsonized with Fc_TFPgp17 and non-opsonized bacteria. Fc_control (Fc-fragment) was used as a control. The results in Fig. 5 for THP-1 macrophages show statistically significant differences in Fc_TFPgp17 1 and 1.5 µM treated samples compared to the untreated control. Similarly, in HL-60 macrophages, we could observe a significant difference in the sample treated with 1.5 µM Fc_TFPgp17 compared to the untreated control (Fig. 6). The increase in MFI event was dose-dependent in both model cell lines. Increased fluorescence signal in TFPgp17 opsonized samples indicates an increased number of bacterial cells engulfed by the phagocytic cells.

### Gentamicin Protection Assay results show fewer viable bacterial cells inside the phagocytic cells compared to the non-opsonized control

**Fig. 7:**
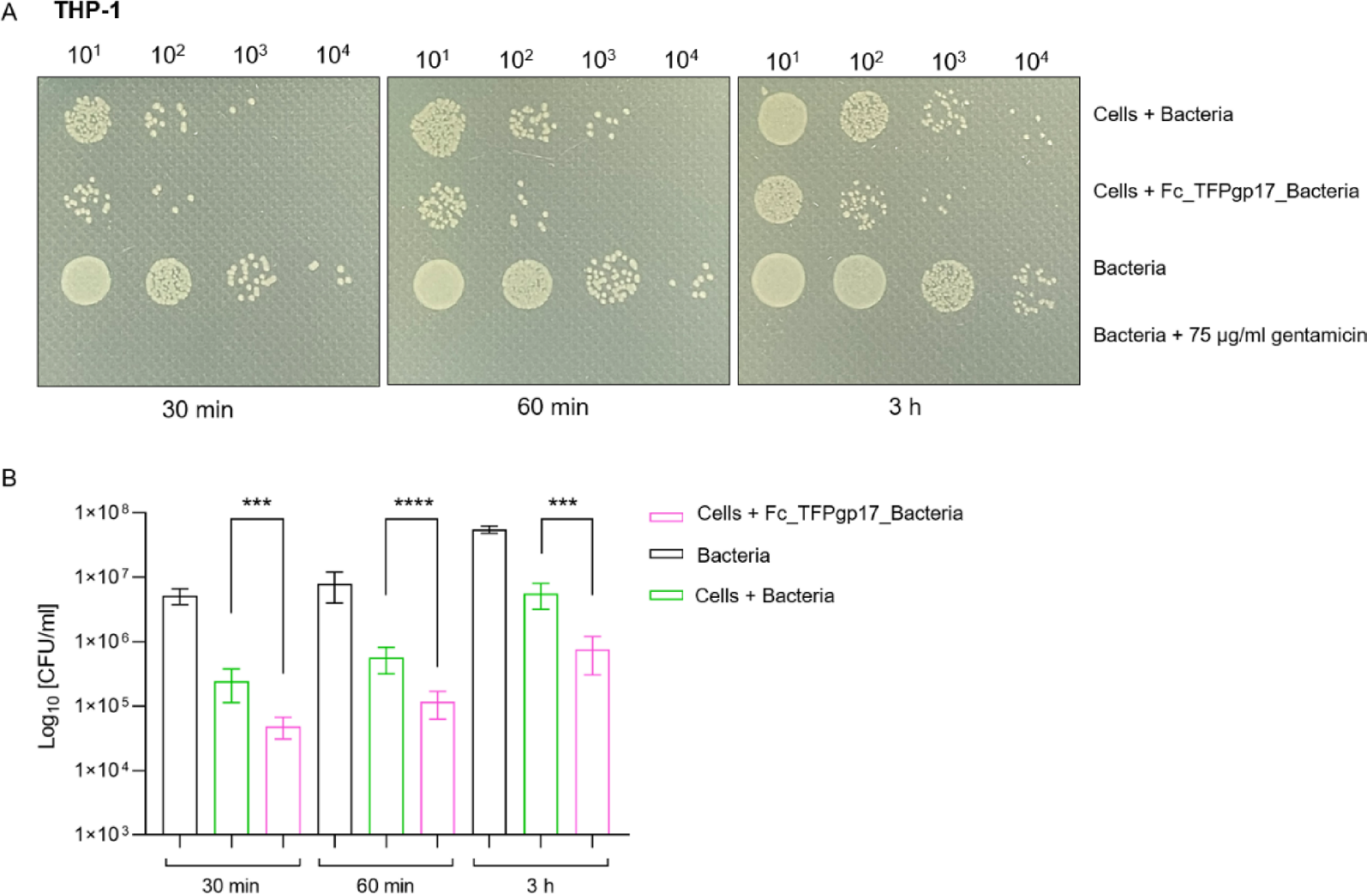
Opsonization with the Fc_TFPgp17 reduces the number of surviving intercellular bacteria in the Gentamicin Protection Assay (GPA) performed using THP-1 macrophages. The figure shows the results of the GPA, conducted at three time points – 30 min, 60 min, and 3 h, using THP-1-derived macrophages. Results demonstrate a significantly lower level of surviving intracellular bacteria in samples where the bacteria were opsonized with Fc_TFPgp17 before the phagocytosis assay, compared to non-opsonized bacteria. Controls included bacteria in the RPMI medium and gentamicin-treated bacteria (negative control). **A** Spot-test results show differences in colony numbers in samples with non-opsonized and Fc_TFPgp17-opsonized bacteria. Serial dilution of samples was spotted on agar plates. **B** The graph illustrates the CFU number of intracellular bacteria in samples with non-opsonized and Fc_TFPgp17-opsonized bacteria. The graph represents data from 2 biological replicates (3 technical replicates). Statistical significance analysis using the unpaired t-test with Welch corrections was performed for the relevant samples (***‑ *P* ≤ 0.001; ****‑ *P* ≤ 0.0001)

**Fig. 8:**
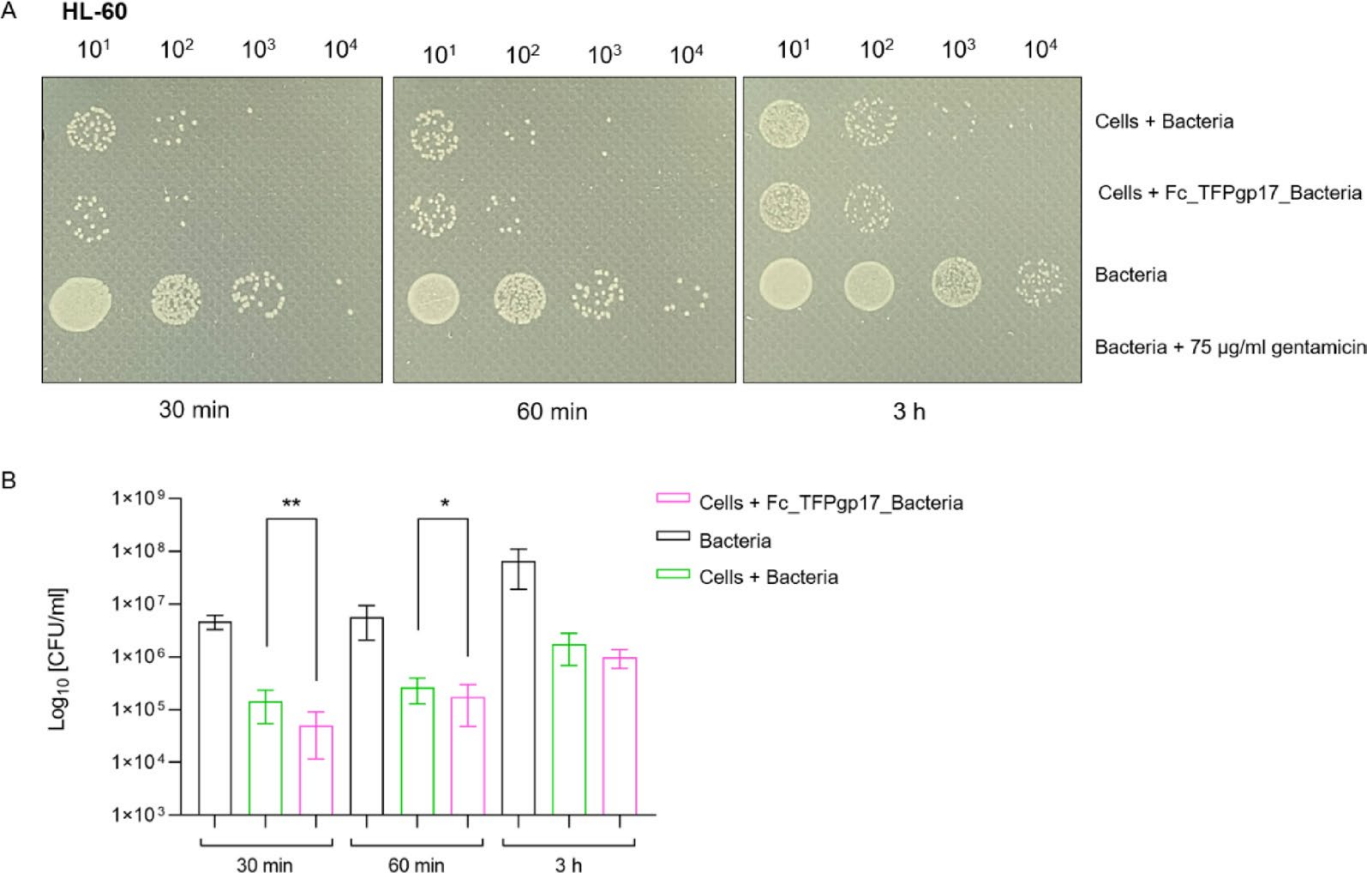
Opsonization with the Fc_TFPgp17 reduces the number of surviving intercellular bacteria in the Gentamicin Protection Assay (GPA) performed using HL-60 neutrophils. The figure shows the results of the GPA, conducted at three time points – 30 min, 60 min, and 3 h, using HL-60-derived neutrophils. Results demonstrate a significantly lower level of surviving intracellular bacteria in samples where the bacteria were opsonized with Fc_TFPgp17 before the phagocytosis assay, compared to non-opsonized bacteria. Controls included bacteria in the RPMI medium and gentamicin-treated bacteria (negative control). **A** Spot-test results show differences in colony numbers in samples with non-opsonized and Fc_TFPgp17-opsonized bacteria. Serial dilution of samples was spotted on agar plates. **B** The graph illustrates the CFU number of intracellular bacteria in samples with non-opsonized and Fc_TFPgp17-opsonized bacteria. The graph represents data from 2 biological replicates (3 technical replicates). Statistical significance analysis using an unpaired t-test with Welch corrections was performed for the relevant samples (*-*P* ≤ 0.05; **‑ *P* ≤ 0.01)

Based on the results obtained in the phagocytosis assay, a 1 µM concentration of Fc_TFPgp17 was chosen for the Gentamicin Protection Assay (GPA). GPA helps to determine the number of surviving bacteria that are protected from gentamicin being intracellular in the lysates of infected cells. Our results proved that increasing the concentration of the Fc_TFPgp17 used for opsonization led to increased amounts of ingested bacteria by phagocytic cells in the flow cytometry assay (Figs. 7 and 8). The next step was to study the intracellular bacteria’s viability and survival rate using GPA. The results of GPA are presented in Fig. 7 for THP-1 and Fig. 8 for the HL-60 cell line. Given the dynamic nature of phagocytosis, we have chosen three time points to study: 30 min, 1 h, and 3 h of incubation. The results obtained in the GPA test in both tested cell lines indicate a lower number of live bacteria in samples where the bacteria were previously opsonized by Fc_TFPgp17 compared to the samples not subjected to opsonization, However, Fc_TFPgp17 was less effective with the HL-60 cell line.

By analysing the results from both flow cytometry and the GPA, it is evident that while Fc_TFPgp17-enhanced opsonization led to increased bacterial uptake by phagocytic cells, the viability of the ingested bacteria was lower compared to the control, where the bacteria were not exposed to Fc_TFPgp17.

### Cytokine level

**Fig. 9:**
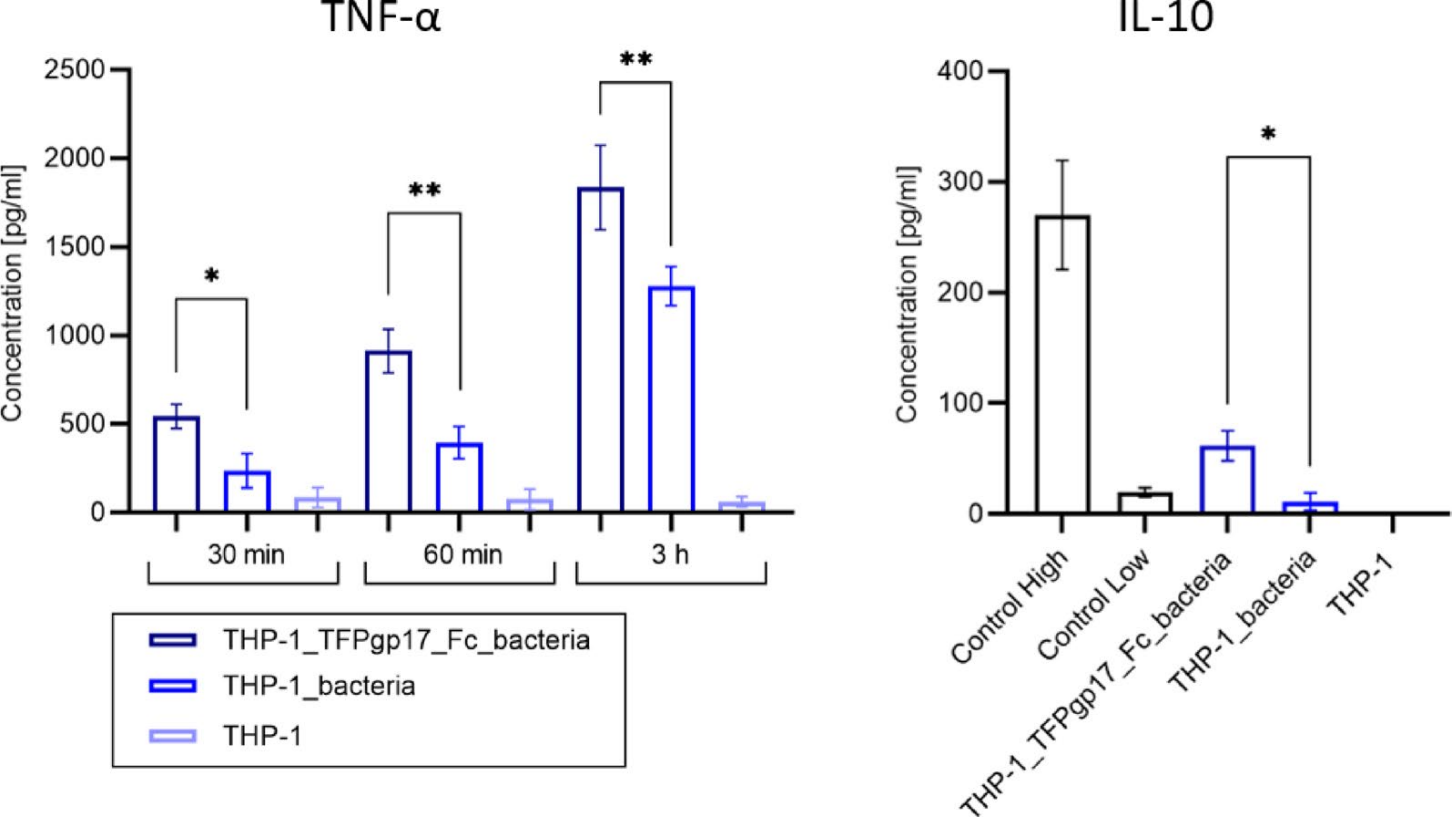
Production of TNF-α and IL-10 was evaluated during the phagocytosis of bacteria opsonized with Fc_TFPgp17 in THP-1 macrophages using commercially available ELISA kits. In the case of TNF-α, three time points were studied because of faster secretion after cell activation. For IL-10, only a 3 h time point is presented in the figure. Statistical significance analysis using an unpaired t-test with Welch corrections was performed for the relevant samples (*-*P* ≤ 0.05; **‑ *P* ≤ 0.01)

The levels of two cytokines were assayed in samples derived from the GPA assay supernatants. We measured the levels of TNF-α and IL-10 for THP-1 as shown in Fig. 9. The pro-inflammatory cytokine TNF-α was measured at three time points during the GPA assay. The results indicate that TNF-α levels increased over time in samples where bacteria were phagocytosed, compared to the negative control, where cells were unstimulated. In samples where bacteria were opsonized with Fc_TFPgp17, TNF-α levels were statistically significantly higher compared to samples with non-opsonized bacteria. Regarding IL-10, an anti-inflammatory cytokine associated with M2 macrophages measured after 3 h of incubation, the results show an increase in production in samples with phagocytosing bacteria, compared to the negative control. Although IL-10 levels did not increase drastically relative to the positive control (control high, provided by the manufacturer as part of the measurement kit), they were higher than those in the low control. Similar to TNF-α, IL-10 levels were higher in samples where Fc_TFPgp17-opsonized bacteria were phagocytosed compared to those with non-opsonized bacteria.

## Discussion

Creating high-affinity therapeutic antibodies for bacterial infections is a non-traditional approach and a significant challenge that warrants further attention and evaluation (Kollef and Betthauser 2021). Monoclonal antibodies (mAbs) have been game-changers in the treatment of cancer, autoimmune diseases, and viral infections, but the development of antibodies for bacterial infections remains problematic (Zurawski and McLendon 2020). Despite the challenges, mAbs present a promising antibacterial strategy with several advantages: (i) longevity, with a typical half-life of 21 days for IgG subclasses; (ii) specificity toward pathogens, which minimizes effects on non-target bacteria in the normal microflora; and (iii) rapid pathogen elimination through multiple mechanisms, including (a) bacterial cell lysis via complement deposition, (b) phagocytosis through bacterial opsonization, (c) toxin neutralization (Martín-Galiano and McConnell 2019; Sawa et al. 2019; Zurawski and McLendon 2020).

Developing antibodies against bacterial surface carbohydrates is challenging due to the high heterogeneity of carbohydrate structures and the increased risk of bacteria developing resistance. Our study proposes a novel solution: a bifunctional protein consisting of an IgG1 Fc fragment fused to a bacteriophage receptor binding protein (RBP). This dual-specific protein functions similarly to an antibody, binding to phagocytic cells via the Fc fragment and to bacterial cells via the phage RBP, thereby promoting opsonization and enhancing phagocytosis by macrophages and neutrophils.

The advantage of this approach lies in the high specificity of phage RBPs toward their receptors on the bacterial surface, which enables fusion proteins to perform similarly to antibodies. Phage tail fibers (TF), located at the distal end of the tail, mediate binding to specific bacterial receptors, such as LPS, porins, teichoic acids, and organelles like pili or flagella. TFs primarily determine host range specificity and ensure effective recognition and adsorption, initiating the infection process. Therefore, utilizing TFs as targeting agents in the development of biologics appears to be a rational and promising strategy. Understanding the interaction between phage TFs and their hosts is an important aspect that will provide essential insights and guidance for developing therapeutics based on phage proteins (Taslem Mourosi et al. 2022). Interestingly, there is evidence in the literature of high structural similarity between the C-terminal fragment of phage RBPs and the variable regions of antibodies (Dunne et al. 2018; Swanson and Cingolani 2018), highlighting their specialization in similar biological functions - the recognition of specific targets.

The initial experiments were performed to confirm the binding of the Fc_TFPgp17 to *Y. enterocolitica* 6471/76-c bacteria and phagocytic cells. We conducted an ELISA assay, which confirmed the binding of the Fc_TFPgp17 to the bacterial cell. However, the binding of the Fc-fusion protein differed from our previous results with the MBP-his tag protein (Filik et al. 2022a). Fc_TFPgp17 was produced in the eukaryotic expression system, specifically in CHO cells, to allow for glycosylation of the Fc fragment, which is essential for binding to Fcγ receptors. We hypothesized that the phage RBP may contain glycosylation sites that lead to a post-translational modification process in CHO cells, which could influence the binding of the Fc_TFPgp17 to its target (Fig. 3C). To test this, a simple experiment was performed in which N-glycans were enzymatically removed from the protein using PNGase F (Fig. 3B). Following N-glycan removal, ELISA was repeated to compare the absorbance of glycosylated and non-glycosylated samples (Fig. 3A). We observed that the absorbance of samples without N-glycans was significantly higher than that of the glycosylated samples.

This suggests that the production of Fc_TFPgp17 in a mammalian expression system leads to glycosylation of the TFPgp17 phage protein, resulting in weaker binding to bacterial cells. One approach to overcome this issue would be to produce Fc_TFPgp17 in bacterial cells, thereby avoiding glycosylation. Additionally, introducing mutations in the Fc fragment could allow the binding of aglycosylated molecules to Fcγ receptors, retaining the effector functions of the molecule (Rashid 2022).

For the Fc_control protein (the Fc fragment fused without TFPgp17), interestingly, this protein binds to *Y. enterocolitica* 6471/76-c bacteria, although the binding was weaker compared to Fc_TFPgp17. Removal of N-glycans resulted in a twofold reduction in absorbance. These results suggest that *Y. enterocolitica* 6471/76-c expresses proteins that can non-specifically bind Fc fragments and that this binding is, at least in part, dependent on the presence of N-glycan residues. It is well known that many bacteria express proteins capable of non-specific binding to immunoglobulins via the Fc fragment as a strategy to evade the host immune response. Prominent examples include protein A from *Staphylococcus aureus* and protein G from group G streptococci (Nordenfelt et al. 2012). Fc-binding proteins have been identified in other pathogenic bacteria such as *Pseudomonas maltophilia* (Grover et al. 1991), *Yersinia pestis* (Zav’yalov et al. 1996), and *Escherichia coli* (Leo and Goldman 2009). There is no data pointing to presence of such protein in *Y. enterocolitica*, but it is highly probable as a member of the *Enterobacteriaceae* family it expresses such proteins. There are examples of *Y. enterocolitica* proteins binding C4bp complement component: YadA and Ail, as a strategy to modulate the immunological response (Kirjavainen et al. 2008).

In our research, we investigated the efficacy of phagocytosis in the presence of the Fc_TFPgp17 molecule (structure shown in Fig. 2). For the in vitro study of phagocytosis, we selected two cell lines: THP-1 (a monocyte cell line isolated from an acute monocytic leukemia patient) and HL-60 (promyeloblasts isolated from peripheral blood). Presented cells do not recapitulate the primary immune cells response, however, they were utilized in the preliminary research presented here as they are best studied models for the process of phagocytosis, well validated in vast range of assays and offer great reproducibility of results. Both cell lines were differentiated for the experiments, THP-1 into macrophages (IL-4 phenotype, M2) and HL-60 into neutrophil-like cells. We used THP-1 and HL-60 because they are widely used models in immunological studies, and extensive literature supports their application in research. This research was designed to establish a model for our preliminary study. Based on the promising results obtained, we plan to expand our work by validating the findings with primary cell lines and PBMCs, and subsequently advancing to in vivo studies.

Our results demonstrate that phagocytosis was significantly more efficient in the presence of Fc_TFPgp17. Flow cytometry data (Figs. 5 and 6) showed that opsonization with 1.5 µM Fc_TFPgp17 resulted in a twofold increase in fluorescence signal compared to the untreated control, indicating that a greater number of bacteria were ingested when opsonized with Fc_TFPgp17. Additionally, the use of pHrodo Green dye provided a distinct advantage, as fluorescence is only emitted once bacteria have been internalized and the intracellular environment becomes acidic, in such conditions as are present in the phagolysosomes. This ensures that the observed fluorescence is specific to bacteria that have been phagocytosed, as the bacteria that are outside the cells do not emit fluorescence signal.-. Thus, higher fluorscene signal indicates increased phagocytic activity of cells – a more acidic environment, caused by phagolysosome formation, is likely related to higher killing activity.

To validate the flow cytometry results, we performed the Gentamicin Protection Assay (GPA), a quantitative method for studying bacterial invasiveness and survival. In this assay, cells are infected with bacteria in vitro, and extracellular bacteria are killed by gentamicin, a bactericidal agent that cannot penetrate the cytoplasm or penetrates it very slowly (Tabrizi and Robins-Browne 1993). Survived intracellular bacteria, protected from gentamicin, are recovered by lysing the infected cells and enumerated by determining colony formation on agar plates. A lower number of surviving bacteria between the tested and control samples indicates a defect in the invasion or intracellular survival of the pathogen (Sharma and Puhar 2019). The dynamics of phagocytosis were examined at three time points, and based on flow cytometry results, we selected 1 µM Fc_TFPgp17 for the assay (Figs. 7 and 8), as this concentration led to nearly twice as many phagocytosed bacteria compared to non-opsonized bacteria in both cell lines.

Notable findings were obtainedregarding the number of surviving bacteria. *Y. enterocolitica* is a difficult model to study because of the ability to multiplication inside the macrophages. In our research we used the strain 6471/76-c which lacks the pYV virulence plasmid responsible for the bacteria invasiveness as well as intacellular survival which results in lower pathogenicity. In both tested cell lines, statistically significant differences were observed: fewer bacteria were present inside phagocytes in samples where bacteria were opsonized with Fc_TFPgp17 compared to phagocytosis of non-opsonized bacteria at every time point (Figs. 7 and 8). This suggests that in these samples, a portion of the bacteria had already been inactivated, for example through oxygen-dependent and independent killing mechanisms. Conversely, bacterial numbers increased over time, this observation can be explained by the fact that phagocytic cells continuously ingest and eliminate bacterial cells. Flow cytometry analysis confirmed that opsonization markedly enhanced bacterial uptake.

In our research, we also measured the level of two cytokines important for macrophages, pro-inflammatory TNF-α, and anti-inflammatory IL-10 which are specific for M2 macrophages. These cytokines are produced by the activated macrophages and play opposite roles in both innate and adaptive immune responses (Shmarina et al. 2001). Immune response to pathogens involves the rapid activation of proinflammatory cytokines that serve to initiate host defense against microbial invasion (Iyer and Cheng 2012). Tumor Necrosis Factor Alpha (TNFα) is a cytokine released during the inflammation caused by infections. The different TNF cell death checkpoints appear to have evolved as a response of the host to this microbial invasion. TNF binding to TNF receptor 1 (TNFR1) directly signals to establish an inflammatory response, primarily by promoting inflammatory gene activation by the MAPK and NF-κB signaling pathways, which leads to cytokine and chemokine release and immune cell recruitment. Activation of TNFR1 indirectly promotes inflammation by triggering cell death. Loss of membrane integrityleads to lytic forms of death - including apoptosis-driven secondary necrosis, pyroptosis, necroptosis - and release of DAMPs (damage-associated molecular patterns), which trigger activation of pro-inflammatory gene expression by bystander cells. Bystander cells can release cytokines and interferons and increase immune cell recruitment (Van Loo and Bertrand 2023), overzealous production of TNF may have serious adverse effects, such as tissue damage and septic shock. The primary function of TNF is to activate and recruit additional immune cells to the site of infection. Macrophages are major producers of TNF-α and are also highly responsive to it. TNF-α levels are frequently measured following phagocytosis to assess macrophage activation. Our results indicate that macrophage activation and subsequent TNF-α secretion may enhance the antimicrobial activity of these cellsIn our study, the level of TNF-alpha was higher in samples where bacteria, before phagocytosis, were opsonized with Fc_TFPgp17. These findings suggest that the cells more effectively recognized the bacterial cells, as indicated by the elevated TNF-α production. TNF-α can promote local inflammation, potentially enhancing the immune response to pathogens. Based on this, increased TNF-α levels may also be associated with enhanced phagocytic activity. This is a promising finding and demonstrates that opsonization with an Fc_TFP molecule—an antibody-mimicking molecule—effectively induces macrophage activation (Fig. 9). As previously mentioned, the second cytokine studied was IL-10. We differentiated the THP-1 cell line with IL-4, which promoted the development of the M2 macrophage phenotype. IL-10 is a potent anti-inflammatory cytokine that plays a central role in limiting the host immune response to pathogens, thereby preventing host tissue damage and maintaining homeostasis (Iyer and Cheng 2012). IL-10 has also a regulatory role in phagocytosis - it does not directly drive the phagocytosis of pathogens for clearance, but instead regulates the process. Up-regulation of IL-10 is beneficial in the early phase of infection because it limits the extent of the immune response, prevents excessive inflammation and immune-mediated damage, and allows inflammation resolution after pathogen elimination (Carlini et al. 2023). What is important, rapid increase in TNF-α is counterbalanced by early and sustained expression of anti-inflammatory IL-10. In our study, IL-10 levels were measured after 3 h, as IL-10 is not produced as rapidly as TNF-alpha following cell activation due to phagocythosis. Our results show that macrophages stimulated by the opsonized bacteria by bispecific Fc_TFPgp17 exhibit a higher level of IL-10 than cells stimulated by the pathogen without opsonization. Similarly to TNF-α, the higher level of IL-10 is implicated with the higher phagocytic activity. In addition, elevated IL-10 levels can result from engagement of Fcγ receptors on immune cells which is in agreement with the proposed mode of action of our molecule.

There is a growing rationale for the development of alternative antimicrobial therapies, which could be both antimicrobial and prophylactic. Many pathogens are becoming resistant to standard antibiotic therapy, and doctors are beginning to have fewer and fewer options to help combat life-threatening infections in patients. The first attempt to use endolysin binding motifs to create Fc-modified proteins showed the potential of bacteriophage proteins to create therapeutic antibodies (Raz et al. 2017, 2018). Our approach to using RBP properties to design Fc-fused antibody-mediated proteins seems to be very promising and new. Previously, phage RBP was only investigated for use as a diagnostic tool, and our approach is the first attempt to use bacteriophage RBP/TFP as a therapeutic. A major advantage of this approach is undoubtedly the natural ability of these proteins to bind receptors on the surface of bacterial cells as a result of their biological properties - initiation of bacteriophage infection through adhesion to specific receptors on the host. In summary, our approach demonstrates the feasibility of developing therapeutic antibodies based on bacteriophage tail proteins. Our results show that RBP in fusion with Fc could provide a new research direction to develop a treatment strategy using therapeutic antibodies based on bacteriophage proteins with high specificity against bacterial pathogens, especially bacterial cell wall carbohydrates. The use of such an approach could contribute to the development of effective therapies for the treatment of life-threatening bacterial infections, to strengthen the natural response of the immune system.

## Conclusion

Fc_TFPgp17 is a bifunctional protein mimicking antibodies that possess two biological functions: (I) recognition of *Y. enterocolitica* O:3 cells via phage RBP (TFPgp17) and (II) recognition of phagocytic cells via Fc-fragment IgG1 antibody. We demonstrated that the modified protein causes more effective phagocytosis of the pathogen by opsonizing bacterial cells, which enables faster recognition of the pathogen and its subsequent phagocytosis. Bacteria treated with Fc_TFPgp17 are more visible to phagocytic cells, effectively recognized and engulfed by phagocytic cells. Increased levels of two key cytokines, pro-inflammatory TNF-α and anti-inflammatory IL-10, indicate phagocytic cell activation which could contribute to more efficient pathogen clearance.

## Supplementary Information

Below is the link to the electronic supplementary material.

Supplementary Material 1

## Data Availability

The datasets used and/or analyzed during the current study are available from the corresponding author upon reasonable request.
