# Supplementary material for: Phage receptor binding protein and Fc fragment fusion enhances phagocytosis of Y. enterocolitica
Source: AMB Express. 2025 Sep 26;15:135. doi: 10.1186/s13568-025-01948-9 (PMC12474815; doi:10.1186/s13568-025-01948-9)
Supplement: Supplementary file 1 — Supplementary Material 1 [file 13568_2025_1948_MOESM1_ESM.docx]

Supplementary materials

# Phage receptor binding protein and Fc fragment fusion enhances phagocytosis of *Y. enterocolitica*

# Karolina Filik-Matyjaszczyk^1,*^, Irwin Matyjaszczyk^2^, Marzena Ciesielska^1^, Bożena Szermer-Olearnik^1^, Krzysztof Mikołajczyk^1^, Andrzej Gamian^1^

^1^ Hirszfeld Institute of Immunology and Experimental Therapy, Polish Academy of Sciences, Wroclaw, 53-114, Poland

^2^Selvita S.A., Department of Biochemistry, Cracow, 30-394, Poland

[^*^karolina.filik@hirszfeld.pl](mailto:*karolina.filik@hirszfeld.pl)

**Materials and methods:**

Genetic construct design:

N-terminal fusion Fc_TFPgp17

MHSSALLCCLVLLTGVRAMATTIKTVMTYPLDGSTTDFNIPFEYLARKFVRVTLIGVDRKELILNQDYRFATKTTISTTRALGPADGYTLIEIRRFTSATDRLVDFTDGSILRAYDLNISQVQTLHVAEEARDLTADTIGVNNDGNLDARGRRIVNVADAQDVGDAINLGQIQRWNDSALNSANRAKQEADRATARANDANNSANASASSASSSAGSAELAKRWATSDTVVESDLESSRTYALHSMLYRNETKDSADRAAVSETNAKASEGGAANSAAAAKVSETNAKASEERAITEASKLGNMNDFAAAIESVTGNDVKMKGAVSSPGNITGGGLVSTGAASIQKGALVGEDLIVGRDITAKQDMYSQRNIAVAGVTYAQGGIEQTLATNIYNKLYRLHINSNPQHVGQRQGLHIGWNESGSGESNFITNRGAGSGGFVFRTVNAENSVETGRVDITGGGVIYANHLQVRSGARIEGNNNIVGQNLYAGMGSTMFEGNGNLTGGIWAQWGNLWSGLNNNSLFAKPPGGVQLFTARGGYYLEGRVDGTAVGFRWFQSDRRLKEDIKVVRSADDMLNIIRSYIPVSYKYKDASYTDNRGRTNTIEGKRSRAGFITQDLIRLWPEAVDVMSDGMQSPDPNQIIGGLMLLVKNLDARIQELEKDKTGGGGSGGGGSGGGGSEPKSCDKTHTCPPCPAPELLGGPSVFLFPPKPKDTLMISRTPEVTCVVVDVSHEDPEVKFNWYVDGVEVHNAKTKPREEQYNSTYRVVSVLTVLHQDWLNGKEYKCKVSNKALPAPIEKTISKAKGQPREPQVYTLPPSREEMTKNQVSLTCLVKGFYPSDIAVEWESNGQPENNYKTTPPVLDSDGSFFLYSKLTVDKSRWQQGNVFSCSVMHEALHNHYTQKSLSLSPGK

Signal_peptide
Protein of interest
Linker
Hinge
CH2_CH3

N-teriminal Fusion control

MHSSALLCCLVLLTGVRAGGGGSGGGGSGGGGSEPKSCDKTHTCPPCPAPELLGGPSVFLFPPKPKDTLMISRTPEVTCVVVDVSHEDPEVKFNWYVDGVEVHNAKTKPREEQYNSTYRVVSVLTVLHQDWLNGKEYKCKVSNKALPAPIEKTISKAKGQPREPQVYTLPPSREEMTKNQVSLTCLVKGFYPSDIAVEWESNGQPENNYKTTPPVLDSDGSFFLYSKLTVDKSRWQQGNVFSCSVMHEALHNHYTQKSLSLSPGK

Signal_peptide
Linker
Hinge

CH2_CH3

**Results:**

Supp Figure 1. SEC Chromatograms after protein purification steps and SDS-PAGE analysis provided by Biointron company: (A) TFPgp17. (B) Fc-control


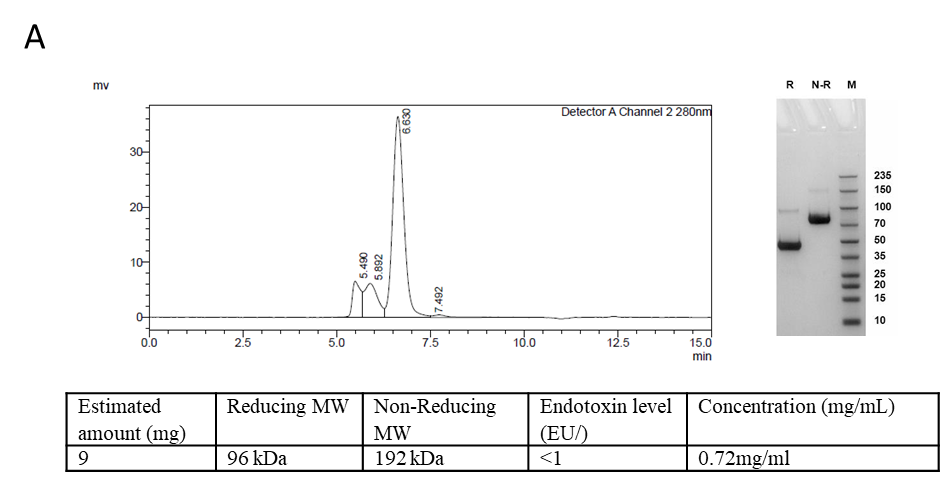


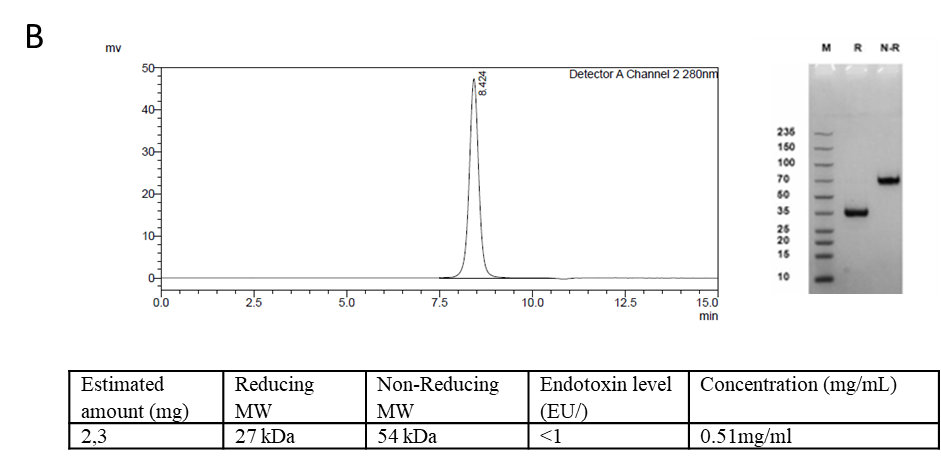


Supp Figure 2. FcγR content in differentiated cells:

1. THP-1-derived macrophages


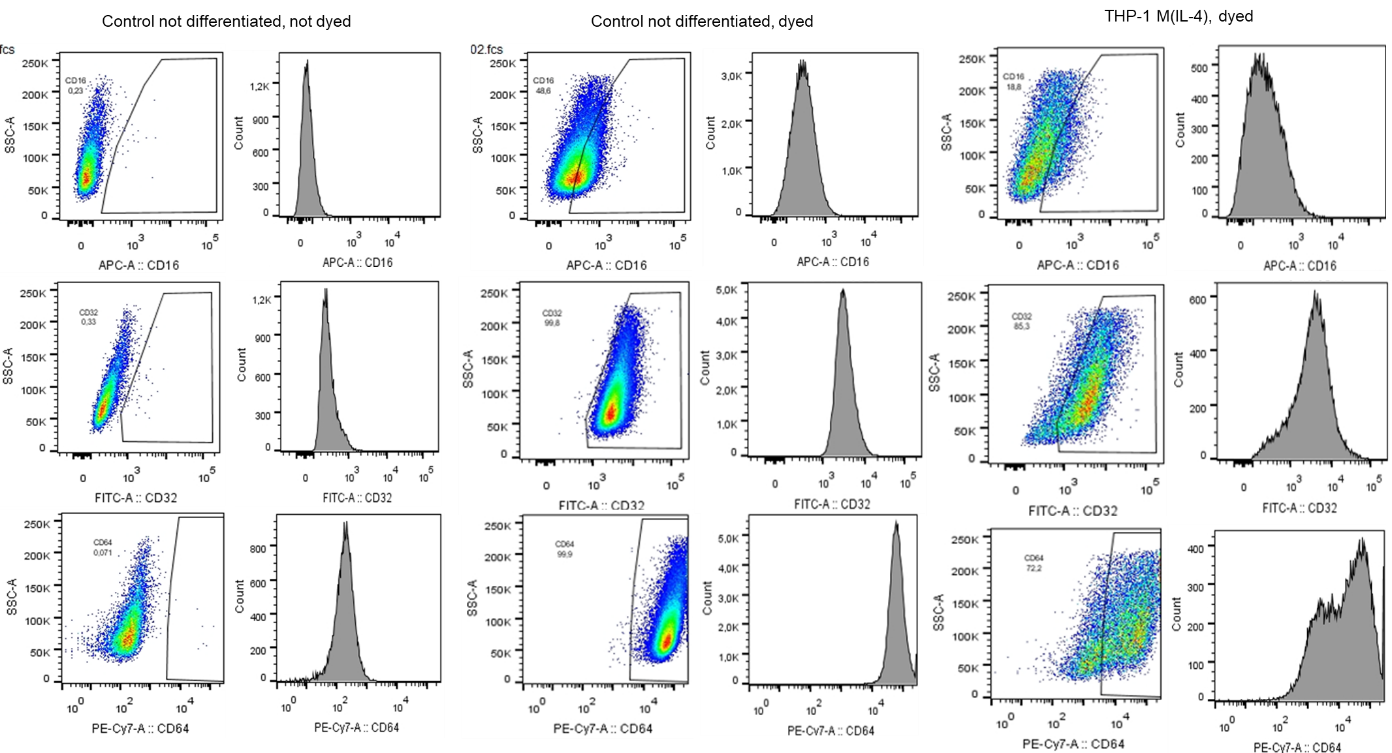


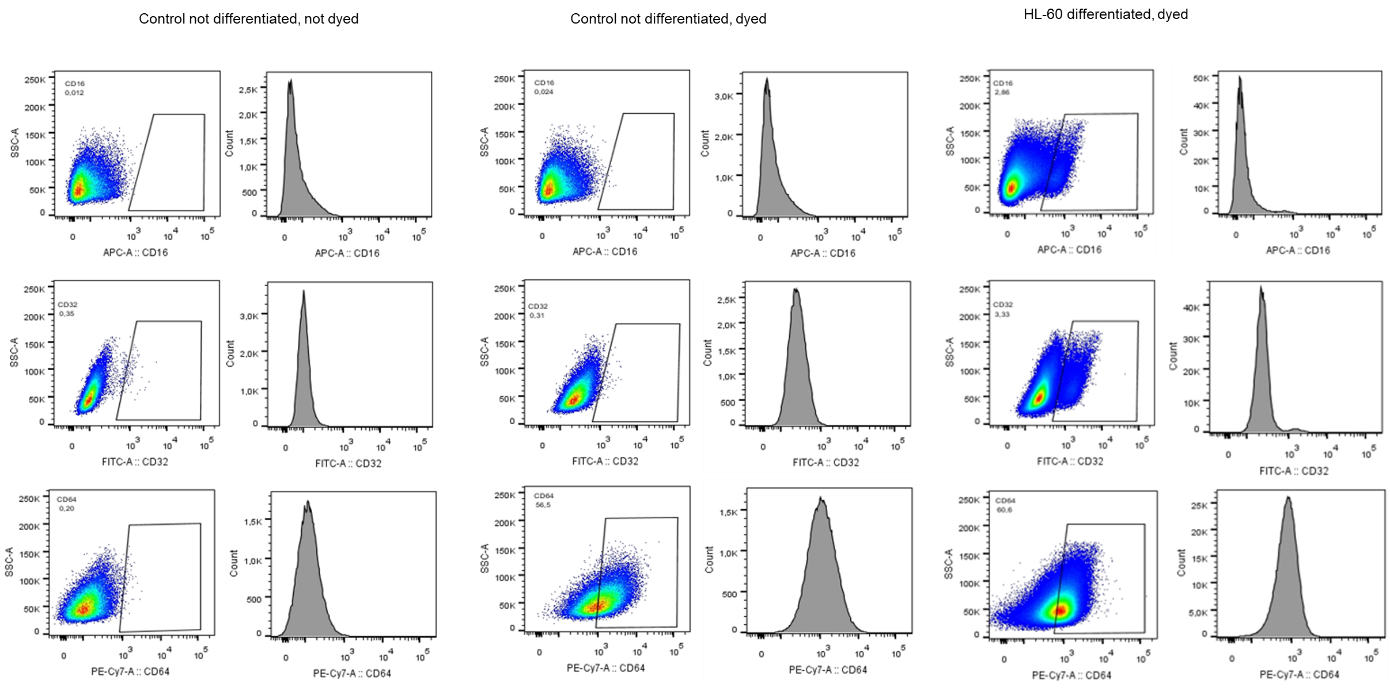


1. HL-60-derived neutrophils
